# Supplementary material for: The Systems Biology Research Tool: evolvable open-source software
Source: BMC Syst Biol. 2008 Jun 29;2:55. doi: 10.1186/1752-0509-2-55 (PMC2446383; doi:10.1186/1752-0509-2-55)
Supplement: Additional file 1 — SBRT Archive. An archive of the current version of the Systems Biology Research Tool. [file 1752-0509-2-55-S1.zip › sbrt-1.4.0/doc/developers_guide/api/sbrt/shell/text/chem/RxnNameFormatV1.html]

RxnNameFormatV1


|  |  |  |
| --- | --- | --- |
| **Overview** \| **Package** \| **Class** \| **Use** \| **Tree** \| **Deprecated** \| **Index** \| **Help** | |  |
| **PREV CLASS**   NEXT CLASS | **FRAMES**    **NO FRAMES**     **All Classes** |  |
| SUMMARY: NESTED \| FIELD \| CONSTR \| METHOD | DETAIL: FIELD \| CONSTR \| METHOD |  |


---


## sbrt.shell.text.chem Class RxnNameFormatV1

```
java.lang.Object
  sbrt.shell.text.chem.RxnNameFormatV1
```

**All Implemented Interfaces:**: RxnNameFormat<java.lang.String>, Format, Formatter<java.lang.String>, Parser<java.lang.String>, SimpleFormat<java.lang.String>

---

``` public final class RxnNameFormatV1 extends java.lang.Object implements RxnNameFormat<java.lang.String> ```

This class is a concrete implementation of `RxnNameFormat`.
This format considers reaction names to be `String` objects.
Reaction names cannot contain any whitespace characters
(spaces, tabs, or newlines) and their length must be greater than 0.

**Author:**
:   This class was written and documented by
    Jeremiah Wright while in the Wagner lab.

---

**Constructor Summary**

`RxnNameFormatV1()`


| **Method Summary** | |
| --- | --- |
| `java.lang.String` | `format(java.lang.String reactionName)`             Returns the provided reaction name after ensuring it conforms to this format. |
| `java.lang.String` | `parse(java.lang.String reactionName)`             Parses the provided string and returns its corresponding reaction name. |

**Methods inherited from class java.lang.Object**

`clone, equals, finalize, getClass, hashCode, notify, notifyAll, toString, wait, wait, wait`

**Constructor Detail**

### RxnNameFormatV1

```
public RxnNameFormatV1()
```


**Method Detail**

### format

```
public java.lang.String format(java.lang.String reactionName)
```

:   Returns the provided reaction name after ensuring it
    conforms to this format.

    :   **Specified by:**: `format` in interface `Formatter<java.lang.String>`
    :   **Parameters:**: `reactionName` - the reaction name to be checked. **Returns:**: the provided reaction name. **Throws:**: `java.lang.IllegalArgumentException` - if the provided reaction name does not conform to this format.: `java.lang.NullPointerException` - if the provided string is `null`.

---


### parse

```
public java.lang.String parse(java.lang.String reactionName)
```

:   Parses the provided string and returns its
    corresponding reaction name.

    :   **Specified by:**: `parse` in interface `Parser<java.lang.String>`
    :   **Parameters:**: `reactionName` - the string to be parsed. **Returns:**: a copy of the provided string with leading and trailing white space removed, or the provided string if it has no leading or trailing white space. **Throws:**: `FormatException` - if the provided string is not a valid reaction name.: `java.lang.NullPointerException` - if the provided string is `null`.


---


|  |  |  |
| --- | --- | --- |
| **Overview** \| **Package** \| **Class** \| **Use** \| **Tree** \| **Deprecated** \| **Index** \| **Help** | |  |
| **PREV CLASS**   NEXT CLASS | **FRAMES**    **NO FRAMES**     **All Classes** |  |
| SUMMARY: NESTED \| FIELD \| CONSTR \| METHOD | DETAIL: FIELD \| CONSTR \| METHOD |  |


---
